# Inappropriate Ventricular Pacing With Exercise in an 11‐Year‐Old Girl With a History of Idiopathic Paroxysmal AV Block

**DOI:** 10.1111/pace.70137

**Published:** 2026-02-09

**Authors:** Collin Kramer, Brock Karolcik, Lee Beerman, Christopher Follansbee, Gaurav Arora

**Affiliations:** ^1^ Division of Pediatric Cardiology The Heart and Vascular Institute UPMC Children's Hospital of Pittsburgh University of Pittsburgh School of Medicine Pittsburgh Pennsylvania USA; ^2^ Department of Pediatrics, Division of Cardiology Texas Children's Hospital and Baylor College of Medicine Houston Texas USA

**Keywords:** idiopathic paroxysmal AV block, pacemaker, pediatric

**Abbreviations**
AV: atrioventricular
BPM: beats per minute
ECG: electrocardiogram

## 1 Clinical Problem

An 11‐year‐old girl presented to establish cardiology care after relocation. She originally presented at 4 years of age with abrupt episodes of syncope associated with abdominal pain, and she was found to have paroxysmal atrioventricular (AV) block of unknown etiology. Her baseline electrocardiogram (ECG) and AV conduction were otherwise normal, and she had a structurally normal heart on echocardiogram. She underwent placement of a single‐chamber epicardial pacemaker (Adapta, Medtronic, Minneapolis, MN, USA). Since that time, she has had done well with no further syncope. Upon presentation to our clinic, the device interrogation revealed the following settings: VVI at 50 beats per minute (BPM), hysteresis at 40 BPM, capture threshold 1.25 V @ 0.4 ms, R waves 16 to >22.4 mV, sensitivity 5.6 mV, impedance 684 ohms with ventricular pacing <0.1%.

**FIGURE 1:**
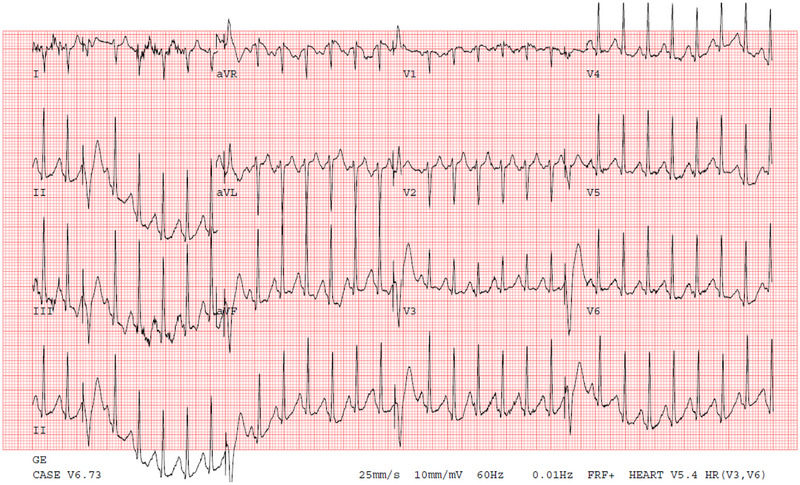
ECG at 181 BPM during stage 3 of treadmill stress test demonstrating occasional ventricular‐paced beats. [Colour figure can be viewed at wileyonlinelibrary.com]

As part of her initial evaluation at our center, the patient underwent an exercise stress treadmill. Her baseline ECG demonstrated sinus rhythm. She was stressed according to the Bruce protocol, and during stage 3, as she reached 181 BPM, she began to have isolated ventricular‐paced beats (Figure 1). Pacing increased in frequency as her heart rate continued to climb to her maximal rate of 200 BPM and continued as she recovered until her heart rate fell to less than 180 BPM before resolving.

*Question for the reader*: What is the cause of the ventricular pacing?

## 2 Commentary

The tracing demonstrates ventricular pacing during intrinsic sinus rhythm with intact AV conduction, which should not occur in VVI mode with appropriate pacemaker function. The differential diagnosis would include undersensing, but in this case, the R waves were robust, and the sensitivity was programmed appropriately. The progressive nature of the pacing, occurring first around 180 BPM, suggested a pacemaker‐determined refractory issue.

**FIGURE 2:**
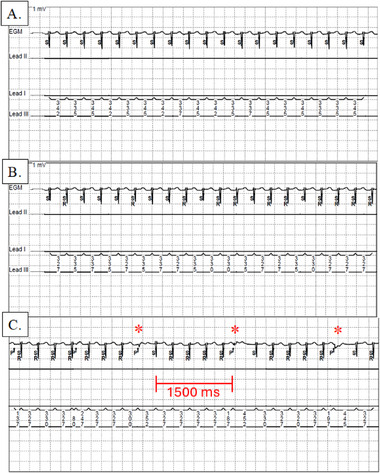
Pacemaker electrograms at 175 BPM (A), 180 BPM (B), and 183 BPM (C). At 175 BPM, there is no ventricular pacing. At 180 BPM, the ventricular refractory period threshold is reached on some beats, but pacing is not yet delivered as subsequent beats have a cycle length > 330 ms and are sensed appropriately. At 183 BPM, there are successive ventricular refractory events which result in a ventricular paced event once the interval reaches 1500 ms (bracketed). [Colour figure can be viewed at wileyonlinelibrary.com]

Her device had a nominal ventricular refractory period of 330 ms or 181 BPM. As her heart rate approached 181 BPM, she had individual beats with cycle length ≤ 330 ms, which initiated ventricular refractory events. Once her heart rate consistently crossed 181 BPM, she had a pattern of repetitive ventricular refractory events and ultimate ventricular pacing when she reached the hysteresis rate of 40 BPM (1500 ms, Figure 2). With reprogramming of the ventricular refractory period to 270 ms, the ventricular pacing was eliminated, and all ventricular events were appropriately sensed.

This behavior occurred due to the combination of single‐chamber pacing with high intrinsic heart rate, as can be seen with devices for pause protection in young patients. While the theoretical risk of inappropriate pacing occurring due to the use of the ventricular refractory period has been described, an actual case has never been reported to our knowledge [1]. Providers should be aware of this possibility to avoid unnecessary and potentially dangerous inappropriate ventricular pacing.

## Author Contributions

C.K. drafted the original article. G.A. participated in the direct patient care and supervised the editing of the article. B.K., L.B., and C.F. all made significant contributions in editing the article.

## Disclosure

The authors have nothing to report.

## Conflicts of Interest

The authors declare no conflicts of interest.

## Data Availability

The data that support the findings of this study are available on request from the corresponding author. The data are not publicly available due to privacy or ethical restrictions.
